# Development and multicenter validation of a multiparametric imaging model to predict treatment response in rectal cancer

**DOI:** 10.1007/s00330-023-09920-6

**Published:** 2023-07-14

**Authors:** Niels W. Schurink, Simon R. van Kranen, Joost J. M. van Griethuysen, Sander Roberti, Petur Snaebjornsson, Frans C. H. Bakers, Shira H. de Bie, Gerlof P. T. Bosma, Vincent C. Cappendijk, Remy W. F. Geenen, Peter A. Neijenhuis, Gerald M. Peterson, Cornelis J. Veeken, Roy F. A. Vliegen, Femke P. Peters, Nino Bogveradze, Najim el Khababi, Max J. Lahaye, Monique Maas, Geerard L. Beets, Regina G. H. Beets-Tan, Doenja M. J. Lambregts

**Affiliations:** 1 Department of Radiology, The Netherlands Cancer Institute, Amsterdam, The Netherlands; 2 GROW School for Oncology & Developmental Biology, University of Maastricht, Maastricht, The Netherlands; 3 Department of Radiation Oncology, The Netherlands Cancer Institute, Amsterdam, The Netherlands; 4 Department of Epidemiology and Biostatistics, The Netherlands Cancer Institute, Amsterdam, The Netherlands; 5 Department of Pathology, The Netherlands Cancer Institute, Amsterdam, The Netherlands; 6 Department of Radiology, Maastricht University Medical Centre, Maastricht, The Netherlands; 7 Department of Radiology, Deventer Ziekenhuis, Schalkhaar, The Netherlands; 8 Department of Interventional Radiology, Elisabeth Tweesteden Hospital, Tilburg, The Netherlands; 9 Department of Radiology, Jeroen Bosch Hospital, ’s-Hertogenbosch, The Netherlands; 10 Department of Radiology, Northwest Clinics, Alkmaar, The Netherlands; 11 Department of Surgery, Alrijne Hospital, Leiderdorp, The Netherlands; 12 Department of Radiology, Spaarne Gasthuis, Haarlem, The Netherlands; 13 Department of Radiology, IJsselland Hospital, Capelle aan den IJssel, The Netherlands; 14 Department of Radiology, Zuyderland Medical Center, Heerlen, The Netherlands; 15 Department of Radiology, Acad. F. Todua Medical Center, Research Institute of Clinical Medicine, Tbilisi, Georgia; 16 Department of Surgery, The Netherlands Cancer Institute, Amsterdam, The Netherlands; 17 Institute of Regional Health Research, University of Southern Denmark, Vejle, Denmark

**Keywords:** Rectal neoplasms, Chemoradiotherapy, Magnetic resonance imaging

## Abstract

**Objectives:**

To develop and validate a multiparametric model to predict neoadjuvant treatment response in rectal cancer at baseline using a heterogeneous multicenter MRI dataset.

**Methods:**

Baseline staging MRIs (T2W (T2-weighted)-MRI, diffusion-weighted imaging (DWI) / apparent diffusion coefficient (ADC)) of 509 patients (9 centres) treated with neoadjuvant chemoradiotherapy (CRT) were collected. Response was defined as (1) complete versus incomplete response, or (2) good (Mandard tumor regression grade (TRG) 1–2) versus poor response (TRG3-5). Prediction models were developed using combinations of the following variable groups:

(1) Non-imaging: age/sex/tumor-location/tumor-morphology/CRT-surgery interval

(2) Basic staging: cT-stage/cN-stage/mesorectal fascia involvement, derived from (2a) original staging reports, or (2b) expert re-evaluation

(3) Advanced staging: variables from 2b combined with cTN-substaging/invasion depth/extramural vascular invasion/tumor length

(4) Quantitative imaging: tumour volume + first-order histogram features (from T2W-MRI and DWI/ADC)

Models were developed with data from 6 centers (*n *= 412) using logistic regression with the Least Absolute Shrinkage and Selector Operator (LASSO) feature selection, internally validated using repeated (*n* = 100) random hold-out validation, and externally validated using data from 3 centers (*n* = 97).

**Results:**

After external validation, the best model (including non-imaging and advanced staging variables) achieved an area under the curve of 0.60 (95%CI=0.48–0.72) to predict complete response and 0.65 (95%CI=0.53–0.76) to predict a good response. Quantitative variables did not improve model performance. Basic staging variables consistently achieved lower performance compared to advanced staging variables.

**Conclusions:**

Overall model performance was moderate. Best results were obtained using advanced staging variables, highlighting the importance of good-quality staging according to current guidelines. Quantitative imaging features had no added value (in this heterogeneous dataset).

**Clinical relevance statement:**

Predicting tumour response at baseline could aid in tailoring neoadjuvant therapies for rectal cancer. This study shows that image-based prediction models are promising, though are negatively affected by variations in staging quality and MRI acquisition, urging the need for harmonization.

**Key Points:**

- *This multicenter study combining clinical information and features derived from MRI rendered disappointing performance to predict response to neoadjuvant treatment in rectal cancer.*
- *Best results were obtained with the combination of clinical baseline information and state-of-the-art image-based staging variables, highlighting the importance of good quality staging according to current guidelines and staging templates.*
- *No added value was found for quantitative imaging features in this multicenter retrospective study. This is likely related to acquisition variations, which is a major problem for feature reproducibility and thus model generalizability.*

**Supplementary information:**

The online version contains supplementary material available at 10.1007/s00330-023-09920-6.

## Introduction

Locally advanced rectal cancer (LARC) is typically treated with neoadjuvant chemoradiotherapy (CRT) followed by surgery [1]. In up to 15–27% of the cases a complete tumor remission is achieved as a result of CRT [2]. This has contributed to the recent paradigm shift in rectal cancer treatment towards organ preservation (e.g., “watch-and-wait” or local treatment of small tumor remnants) for selected patients with clinical evidence of a very good or complete tumour response after CRT. For these organ-preservation approaches, the morbidity and mortality risks associated with major surgery are avoided, with good reported clinical outcomes regarding quality of life and overall survival [3, 4]. Predicting the response to CRT and thus the chance of achieving organ preservation before the start of treatment, i.e., at baseline, may open up new possibilities to further personalize neoadjuvant treatment strategies depending on the anticipated treatment benefit, particularly for smaller tumors that do not necessarily require CRT for oncological reasons.

Recent studies have suggested a possible role for imaging in this setting [5–9]. Promising results have been reported for clinical staging variables (MRI-based TN-stage) [6, 7], tumor volume [10–12], and functional parameters derived from diffusion-weighted imaging (DWI) [8, 9] or dynamic contrast-enhanced MRI (DCE) [13] to predict rectal tumor response on baseline MRI, and more recently also for more advanced quantitative variables derived using modern post-processing tools such as radiomics [5]. However, the available evidence mainly comes from single-center studies and comprehensive multicenter studies incorporating clinical, functional as well as advanced quantitative imaging data are scarce [14, 15]. Moreover, the effects of multicenter data variations and diagnostic staging differences between observers so far remain largely uninvestigated. Prediction studies on larger multicenter patient cohorts with imaging data acquired and analyzed as part of everyday clinical routine are therefore urgently needed to develop a more realistic view of the potential role of image-based treatment prediction models in general clinical practice.

In this retrospective multicenter study, we therefore set out to develop and validate a model to predict response to neoadjuvant treatment in rectal cancer using rectal MRIs acquired for baseline staging in 9 different centers in the Netherlands, intended to be a representative sample of rectal imaging performed in everyday clinical practice.

## Materials and methods

### Patients

**Fig. 1:**
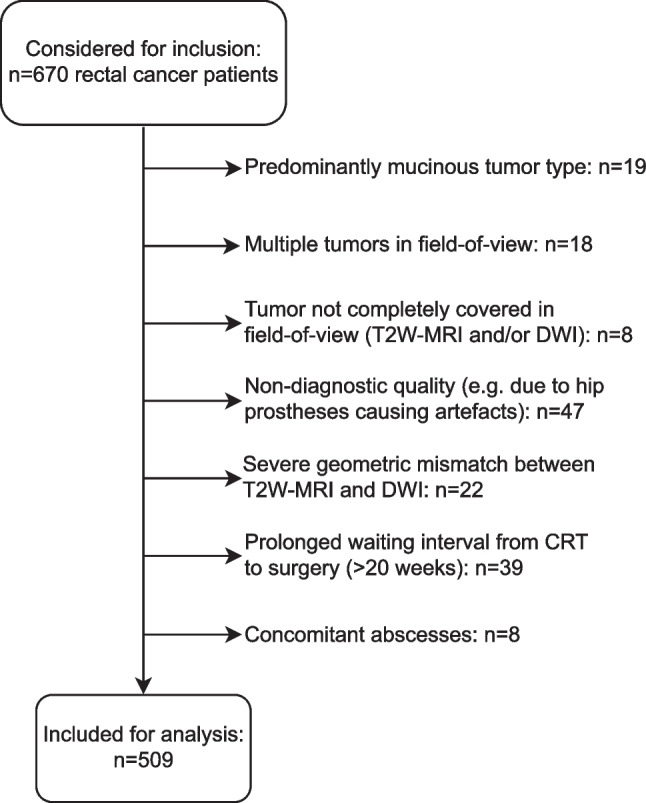
In- and exclusion flowchart. Note, mucinous tumors were excluded because these are known to exhibit distinctly different signal characteristics on both T2W-MRI and DWI

As part of an institutional review board-approved multicenter study project, the clinical and imaging data of 670 LARC patients undergoing standard-of-care neoadjuvant chemoradiotherapy between February 2008 and March 2018 were retrospectively collected from 9 study centers (1 university hospital, 7 large teaching hospitals, and 1 comprehensive cancer center). Patients were identified based on the following inclusion criteria: (a) biopsy-proven rectal adenocarcinoma, (b) non-metastasized disease, (c) availability of a pre-treatment MRI (including at least T2-weighted (T2W) sequences in multiple planes and an axial DWI sequence) with corresponding radiological staging report (d) long-course neoadjuvant treatment consisting of radiotherapy (total dose 50.0–50.4 Gray) with concurrent capecitabine-based chemotherapy, (e) final treatment consisting of surgery or watch-and-wait with >2 years clinical follow-up to establish a reliable final response to CRT. From this initial cohort, 161 patients were excluded for reasons detailed in Fig. 1, leaving a total study population of *n*=509. Due to the retrospective nature of this study, informed consent was waived.

### Imaging and image pre-processing

**Fig. 2:**
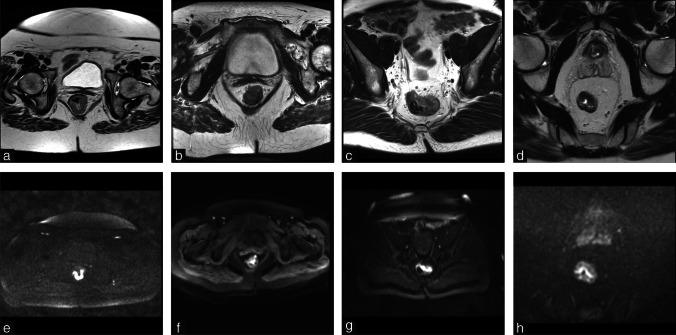
Examples illustrating differences in image quality and acquisition for T2W-MRI (**a**–**d**) and DWI (**e**–**h**) between centers, related to for example field-of-view, tissue contrast (e.g., TR/TE settings), image resolution, and noise. For the DWI scans, the highest acquired b-values shown in these examples were b1000 (**e**), b600 (**f**), b800 (**g**), and b1000 (**h**)

MRIs were acquired according to routine practice in the participating centers with substantial variations in scan protocols and corresponding image quality between and within centers (Fig. 2); images were acquired using 25 different scanners (19 1.5T; 6 3.0T) and a total of 112 unique T2W and 94 unique DWI protocols. Further parameters are summarized in Supplementary Materials A. From the source DW images we calculated the Apparent Diffusion Coefficient (ADC) maps using all available b-values (varying from 2–7 *b* values per sequence; *b* values ranging between b0 and b2000) using a mono-exponential fit. ADC values <0 or >3 standard deviations from the tumor mean were marked as invalid. Since T2W pixel values are represented on an arbitrary scale, these images were normalized to mean=0 and standard deviation=100 [16]. All images were resampled to a common isotropic pixel spacing of 2 mm x 2 mm x 2 mm using a Linear interpolator for the DWI and ADC maps (linear was chosen to prevent out-of-range intensities which may occur due to overshoot with higher order interpolations) and a B-Spline interpolator for T2W images.

### Image evaluation

Baseline staging variables (cT-stage (cT1-2, cT3, cT4), cN-stage (cN0, cN1, cN2), and involvement of the mesorectal fascia (MRF)) were derived from the original staging reports that were performed by a multitude of readers. In addition, all MRIs were retrospectively re-evaluated for the purpose of this study by a dedicated radiologist (DMJL, with >10 years’ experience in reading rectal MRI) who staged all cases in line with the latest staging guidelines and reporting template from the European Society of Gastrointestinal and Abdominal Radiology [17]. For quantitative analysis, tumors were segmented using a 3D slicer (version-4.10.2). Segmentations were acquired semi-automatically using a level-tracing algorithm applied to the high *b* value DWI, which were then manually adjusted by an expert radiologist (DMJL, the same reader who also staged the cases) taking into account the anatomical information from the corresponding T2W-MRI. Care was taken to include only tumor tissue, excluding the rectal lumen and any non-tumoural perirectal tissues. Segmentations were then copied to the ADC-map and T2W-MRI, after which tumor volume and other quantitative features were extracted with PyRadiomics (version-3.0) using a bin-width of 5 (T2W-MRI) and 5x10^-5^ (ADC). This bin width was chosen such that the number of histogram bins was between 30 and 130 [16]. Quantitative features were limited to simpler volume, and first-order features as these have previously been reported to be most reproducible [19–23] and least dependent on acquisition differences between centers [18].

### Variable definitions

Five distinct variable categories were defined:

1. *Non-imaging variables*; including age, sex, basic tumor descriptors from clinical examination and endoscopy (tumor location and basic tumor morphology, e.g., polyp/circular), and the time interval between neoadjuvant CRT and surgery.
2. *Basic image-based staging variables:*
  *(2a) derived from the original reports,* including cT-stage (cT1-2, cT3, cT4), cN-stage (cN0, cN1, cN2), and MRF involvement, that were routinely available from the original staging reports.
  *(2b) derived from expert re-evaluation, including* the same descriptors from 2a, but now derived from the expert re-evaluations.
3. *Advanced image-based staging variables*; including advanced staging descriptors (tumor length, cT-substage (cT1-2; cT3a,b,c,d; cT4a,b), depth of extramural invasion, and extramural vascular invasion (EMVI)) that were not routinely available from the original staging reports but derived from the expert re-evaluations.
4. *Quantitative imaging features*; including tumor volume (extracted directly from the whole-tumor segmentations), and the following first-order features (derived from the pixel values within the tumor on both T2W-MRI and ADC): mean, median, minimum, maximum, variance, mean absolute deviation, range, robust mean absolute deviation, root mean squared, 10^th^ percentile, 90^th^ percentile, energy, entropy, interquartile range, kurtosis, skewness, total energy, and uniformity.

These five variable categories were combined into eight combinations of variable sets for the statistical analysis as detailed in Table 1.

**Table 1:** Variable category definition and variable sets

Variable categories	Features
1. Non-imaging	Age, sex, time between CRT and surgery, tumor morphology (polyp, semicircular, or circular) and tumor height (distal-mid versus proximal-rectosigmoid)
2a. Basic imaging staging (original reports)	cT-stage (cT12, cT3, cT4), cN-stage (cN0, cN1, cN2), involvement of the mesorectal fascia (MRF-, MRF+)
2b. Basic imaging staging (expert re-evaluation)	cT-stage (cT12, cT3, cT4), cN-stage (cN0, cN1, cN2), involvement of the mesorectal fascia (MRF-, MRF+)
3. Advanced imaging staging (expert re-evaluation)	All variables included in 2b (basic imaging staging—expert re-evaluation) + cT-substage (cT12, cT3abcd, cT4ab), extramural invasion depth, EMVI, tumor length
4. Quantitative imaging (derived from T2W-MRI and ADC)	Tumor volume*, mean, median, minimum, maximum, variance, mean absolute deviation, range, robust mean absolute deviation, root mean squared, 10^th^ percentile, 90^th^ percentile, energy, entropy, interquartile range, kurtosis, skewness, total energy, uniformity
Variable sets	
1. Non-imaging only 2. Non-imaging + basic imaging staging (original reports) 3. Non-imaging + basic imaging staging (expert re-evaluation) 4. Non-imaging + advanced imaging staging (expert re-evaluation) 5. Non-imaging + quantitative imaging 6. Non-imaging + basic imaging staging (original reports) + quantitative imaging 7. Non-imaging + basic imaging staging (expert re-evaluation) + quantitative imaging 8. Non-imaging + advanced imaging staging (expert re-evaluation) + quantitative imaging	

* Tumor volume was derived directly from the whole-tumor segmentations

### Response outcome

The final treatment response was defined in twofold [8, 24, 25]:

- *Complete response (CR) versus incomplete response*: CR was defined as a pathological complete response after surgery (pCR; ypT0N0) or a sustained clinical complete response (cCR) without evidence of recurrence on repeated follow-up MRI and endoscopy for >2 years in patients undergoing watch-and-wait. Patients with ypT1-4 disease after surgery were classified as incomplete responses.
- *Good response (GR) versus poor response*: GR included all patients with Mandard’s tumor regression grade (TRG) of 1–2 (total and subtotal regression); patients with TRG of 3–5 (moderate, limited and no regression) were classified as poor responders. Patients with a sustained cCR for >2 years were considered TRG1. If the pathology report did not explicitly mention a TRG score, the complete pathology reports were reviewed with a dedicated gastrointestinal pathologist (P.S. with >8 years of experience) to assign a TRG score retrospectively.

### Statistical analysis

**Fig. 3:**
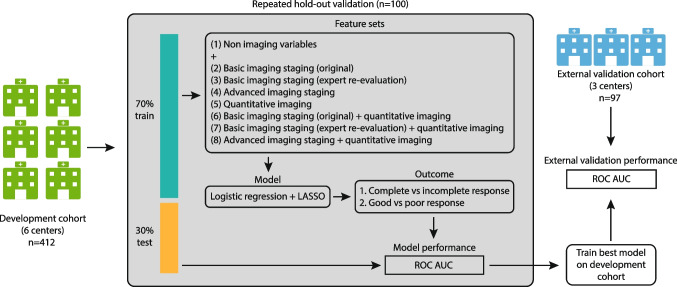
Schematic overview of the study workflow and statistical analysis. From a total cohort of 509 patients from 9 centers, 412 patients (from 6 centers) were used to develop a prediction model to predict two respective outcomes (complete response, good response) using repeated hold-out validation. For both outcomes, the best-performing model was tested on an external and independent validation cohort consisting of 97 patients (from 3 different centers)

The 9 centers were divided into development including 6 centers (*n*=412) and (external) validation set including 3 centers (*n*=97). Differences between development and validation sets were assessed using Chi-squared tests for categorical (sex and response) and Kruskal-Wallis tests for continuous/ordinal variables (age, cTN-stage). The model development and validation process are summarized in Fig. 3. For the eight variable sets (see Table 1) the ability to predict the two respective response outcomes (complete vs incomplete response; good vs poor response) was assessed in the development cohort by calculating the average area under the receiver operator characteristic curve (AUC) after repeated (*n*=100) random hold-out validation. During each iteration, the development cohort was randomly split into a 70% training / 30% test dataset. All training variables were then scaled (mean=0, standard deviation=1), with the same scaling (i.e., using the mean and standard deviation derived from the training set) applied to the test set. When two or more features in a variable set were correlated (with Pearson’s *ρ*>0.8 in the training data), only the feature with the lowest mean absolute correlation was retained for further analysis. The remaining variables were used to train a logistic regression model with the Least Absolute Shrinkage and Selector Operator (LASSO) regularization [26]. The LASSO regularization parameter (*λ*) was tuned to select only the most relevant variables by minimizing the negative binomial log-likelihood loss using internal repeated (*n*=100) 10-fold cross-validation. Each model’s performance was measured on the test dataset, and the model achieving the best average test AUC was trained on the whole development cohort. As a final step, the performance of this best-performing model (N.B. one model for CR and one for GR) was tested on the external validation cohort. 95% confidence intervals for averaged AUCs in the development data were estimated through bootstrapping (200 samples). Confidence intervals for the validation cohort were obtained using DeLong’s method [27].

Supplementary Materials B describes two additional analyses: (1) testing the effects of 3 different previously described methods for multicenter data normalization (using a reference organ [28], statistical correction of imaging features using the ComBat algorithm [29], and statistical correction using mixed-effects models [30]), and (2) comparing model performance in the multicenter dataset to a single-center data subset from the cohort acquired with a harmonized MRI acquisition protocol. The latter was done to mimic the comparison of our results with a single-center study design.

## Results

### Patients

Baseline patient information is presented in Table 2; 332 (65%) patients were male; the median age was 65 years. For the outcome complete (versus incomplete) response, 141 patients (28%) were classified as complete responders. For the outcome good (versus poor) response, 225 patients (44%) were classified as good responders. The development and validation cohort showed no significant differences in sex, age, cT-stage, cN-stage, and tumor response (*p*=0.37–0.98).

**Table 2:** Baseline patient characteristics and variations between centers

		Total	Development cohort	Validation cohort	*p* value
Total, *n* (%)		*n* = 509 (100%)	*n* = 412 (81%)	*n* = 97 (19%)	
Sex, *n* (%)	Female	177 (35%)	139 (34%)	38 (39%)	0.37*
	Male	332 (65%)	273 (66%)	59 (61%)	
Age, median (range)		65 (25-87)	66 (25-87)	65 (33-81)	0.37**
cT, *n* (%)	1-2	35 (7%)	30 (7%)	5 (5%)	0.57**
	3	441 (81%)	334 (81%)	80 (83%)	
	4	60 (12%)	48 (12%)	12 (12%)	
cN, *n* (%)	0	68 (13%)	52 (13%)	16 (17%)	0.98**
	1	122 (24%)	103 (25%)	19 (20%)	
	2	319 (63%)	257 (62%)	62 (64%)	
Complete response, *n* (%)	CR	141(28%)	111 (27%)	30 (31%)	0.51*
	Not-CR	368 (72%)	301 (73%)	67 (69%)	
Good response, *n* (%)	Good	225 (44%)	184 (45%)	41 (42%)	0.75*
	Poor	284 (56%)	228 (55%)	56 (58%)	

*Calculated using chi-squared test

** Calculated using the Kruskal-Wallis test

### Model performance and predictive variables

Results for model development and performance are detailed in Table 3. The best-performing model included non-imaging and advanced imaging staging variables and achieved an average AUC of 0.60 (95%CI 0.48–0.72) to predict a complete response and an AUC of 0.65 (95% CI 0.53–0.76) to predict a good response in the external validation cohort, results very similar to those obtained during testing in the development cohort. The addition of quantitative imaging features did not improve predictive performance in any of the model combinations. Basic staging variables consistently achieved lower predictive performance compared to the advanced staging variables, especially (though 95% confidence intervals showed some overlap) when the basic staging variables were derived from the original reports. Based on the model coefficients, a more proximal tumor location, shorter tumor length, longer waiting interval after CRT, lower cT-substage and cN-stage, negative MRF, lower extramural invasion depth, and negative EMVI status were associated with a favorable response outcome (full model coefficients are provided in Supplementary Materials C).

**Table 3:** Model performance

Variable groups and combinations	Outcome	
	CR (95% CI)	GR (95% CI)
Average AUC on the development cohort		
Non-imaging	0.58 (0.49–0.66)	0.53 (0.42–0.58)
Non-imaging + basic imaging staging (original reports)	0.63 (0.55–0.70)	0.52 (0.39–0.54)
Non-imaging + basic imaging staging (expert re-evaluation)	0.66 (0.58–0.70)	0.62 (0.56–0.68)
Non-imaging + advanced imaging staging (expert re-evaluation)	**0.69** **(0.62**–**0.74)**	**0.67** **(0.62**–**0.73)**
Non-imaging + quantitative imaging	0.59 (0.46–0.61)	0.58 (0.47–0.61)
Non-imaging + basic imaging staging (original reports) + quantitative imaging	0.59 (0.44–0.60)	0.57 (0.44–0.59)
Non-imaging + basic imaging staging (expert re-evaluation) + quantitative imaging	0.63 (0.51–0.68)	0.62 (0.53–0.68)
Non-imaging + advanced imaging staging (expert re-evaluation) + quantitative imaging	0.68 (0.59–0.71)	0.67 (0.61–0.72)
Performance of best-performing model on the external validation cohort		
Non imaging + advanced imaging staging (expert re-evaluation)	0.60 (0.48–0.72)	0.65 (0.53–0.76)
Features selected in CR model: [Intercept], tumor height, weeks to surgery, cTsub-stage, cN-stage, invasion depth (mm), tumor length (mm)		
Features selected in GR model: Tumor height, weeks to surgery, cTsub-stage, MRF-status, invasion depth (mm), EMVI status		

The best-performing models are depicted in bold

*95% CI*, 95% confidence interval; *CR*, complete response (pCR and cCR); *GR*, good response (TRG1-2); *NB*, confidence intervals on the development cohort AUC are based on the non-studentized pivotal bootstrap method^31^ using 200 bootstrap samples. For the external validation cohort, DeLong’s method^27^ was used

The results of Supplementary Materials B show that none of the normalization methods applied to retrospectively harmonize the data led to improved predictive performance. When mimicking a single-center study design (i.e., when performing the same analysis on a single-center subset within our cohort with homogeneous imaging protocols), results were highly variable but showed a trend towards better single-center model performance for most variable subsets to predict a complete response. The best-performing single-center model (including non-imaging and advanced staging variables) achieved an AUC of 0.79, compared to an AUC of 0.69 in the total multicenter development cohort.

## Discussion

This multicenter study shows that when combining clinical baseline variables with image-based staging quantitative variables, overall model performance to predict neoadjuvant treatment response in rectal cancer is disappointing, with externally validated AUCs ranging between 0.60 and 0.65 to predict either a complete response (ypT0) or a good response (TRG1-2). Best model performance was achieved when combining clinical baseline information (e.g., time to surgery) and image-based staging variables (e.g., cT-stage). Quantitative imaging features had no added value. Notably, model performance was considerably better when including modern staging parameters such as cT-substage, extramural invasion depth, and EMVI, compared to more traditional staging including only simplified cTN-stage and MRF involvement. Moreover, model performance seemed to be affected by staging variations between observers with better performance when staging was performed by a dedicated expert compared to the original staging reports acquired by a multitude of readers.

Previous studies typically included staging variables such as cTN-stage as part of the “baseline patient variables,” which implies that these are “objective” variables with little variation between readers [31, 32]. While measurement variations are commonly considered when analyzing quantitative imaging data, our results demonstrate that interobserver variation is also an important issue to take into account for the more basic staging variables. The improved model performance when including also modern staging variables such as cT-substage and EMVI in the expert re-evaluations further highlights the importance of high-quality diagnostic staging using up-to-date guidelines. The clinical impact of ‘state-of-the-art’ staging was also demonstrated by Bogveradze et al, who showed in a retrospective analysis of 712 patients that compared to “traditional” staging methods, advanced staging according to recent guideline updates would have led to a change in risk classification (and therefore potentially in treatment stratification) up 18% of patients [33]. The fact that our cohort dates back as far as 2008 and covers a 10-year inclusion period explains why many of these advanced staging variables could not be derived from the original reports. The use of older data will likely also have impacted the quality of the images and thus the quantitative imaging features derived from the data. Following developments in acquisition guidelines and software and hardware updates, the image quality will have evolved over time. This is also reflected by the large number of different imaging protocols including 112 unique T2W and 94 unique DWI protocols. The question, therefore, remains if and how model performance would have improved using only state-of-the-art and/or more harmonized (prospectively acquired) MRI data. In our current dataset, quantitative imaging features showed no added benefit to predict response. This contradicts previous single-center and smaller bi- and tri-institutional studies that achieved more encouraging AUCs ranging from 0.63 to as high as 0.97 [5, 14, 15]. These previous results are likely at least in part an overestimation of how such models would perform in everyday practice, as especially earlier pilot studies are hampered by limitations in methodological design (e.g., small patient cohorts, re-using of training data for testing, and multiple testing) as also outlined in several review papers reporting on the quality and/or reproducibility of image biomarker studies [5, 19, 34–38]. The fact that most previous studies have been single-center reports will likely have also played an important role. Though reflective of data acquired in everyday practice, our results confirm the known difficulties of building generally applicable prediction models using heterogeneous retrospectively collected multicenter data. While some data variations are necessary to identify robust features to vendor and acquisition differences, too much variation will negatively impact model generalizability. Attempting to directly compare and investigate the effects of multicenter (heterogeneous) versus single-center (homogeneous) modelling using our own data, we mimicked a single-center comparison by repeating our study analyses on a homogeneous single-center subset within our cohort. Though results have to be interpreted with caution considering the wider confidence intervals and lack of external validation in the single-center arm, this comparison suggests that the best-performing model indeed appeared to be better for the homogeneous single-center subset (AUC 0.79) than for the multicenter (AUC 0.69) cohort. Though full data harmonization will likely never be achieved in daily clinical practice, these findings do support a need for further protocol guidelines and standardization to benefit future multicenter research.

There are some limitations to our study design. As mentioned above, data was acquired over the time span of a decade including scans acquired using outdated protocols dating back as far as 2008. A detailed analysis of the impact of these spectrum effects was outside the scope of this study, but a preliminary analysis (results not reported) showed that the impact of temporal changes was negligible. All segmentations were performed on high b-value DWI and then copied to T2W-MRI and ADC maps. Although care was taken to include anatomical information from T2W-MRI during segmentation, ideally a separate segmentation would have been performed. Finally, the comparison between the original basic staging reports and the advanced staging performed as part of this study was influenced by the fact that all re-evaluations were done by a single reader. In contrast, original staging reports were performed by a multitude of readers with varying levels of expertise. Due to the time-consuming nature of the expert re-evaluations (and segmentations), it was unfortunately not deemed feasible to include an independent extra reader.

In conclusion, this multicenter study combining clinical information and MRIs acquired as part of everyday clinical practice over the time span of a decade rendered disappointing performance to predict response to neoadjuvant treatment in rectal cancer. The best results were obtained when combining clinical baseline information with state-of-the-art image-based staging variables, highlighting the importance of good quality staging according to current guidelines and staging templates. No added value was found for quantitative imaging features in this multicenter retrospective study setting. This is likely at least in part the result of acquisition variations, which is a major problem for feature reproducibility and thus model generalizability. To benefit from quantitative imaging features—assuming a predictive potential—further optimization and harmonization of acquisition protocols will be essential to reduce feature variation across centers. For future research, it would also be interesting to see how model performance may improve when combining the information that can be derived from imaging with other clinical biomarkers such as molecular markers (e.g., DNA mutations, gene expression, microRNA) [39, 40], blood biomarkers (e.g., CEA, circulating tumor DNA) [39, 41], metabolomics (e.g., metabolites, hormones, and other signaling molecules) [42], organoids [43], and immune profiling [44].

### Supplementary information

ESM 1 (DOCX 44 kb)

## Abbreviations

ADC: Apparent diffusion coefficient
AUC: Area under the receiver operator characteristic curve
cCR: Clinical complete response
CR: Complete response
CRT: Chemoradiotherapy
DCE: Dynamic contrast-enhanced
DWI: Diffusion-weighted imaging
EMVI: Extramural vascular invasion
GR: Good response
LARC: Locally advanced rectal cancer
LASSO: Least Absolute Shrinkage and Selector Operator
MRF: Mesorectal fascia
MRI: Magnetic resonance imaging
pCR: Pathological complete response
T2W: T2-weighted
TRG: Tumor regression grade

## Declarations

### Guarantor

The scientific guarantor of this publication is Doenja Lambregts, The Netherlands Cancer Institute, Amsterdam, The Netherlands.

### Statistics and biometry

One of the authors (Sander Roberti) has significant statistical expertise.

### Study subjects or cohorts overlap

Some study subjects or cohorts have been previously reported in the following:

*Schurink NW, van Kranen S, Roberti S, et al (2022) Sources of variation in multicenter rectal MRI data and their effect on radiomics feature reproducibility. Eur Radiol. 32:1506-1516.*

*Bogveradze, N., El Khababi, N., Schurink, N. W., et al (2022). Evolutions in rectal cancer MRI staging and risk stratification in The Netherlands. Abdominal radiology, 47(1):38–47.*

The aims of these publications are different than the current novel study.

The first study investigates the influence of acquisition variation on imaging features. The lessons from this study were used and applied in this study to develop response prediction models.

The second study describes how radiology staging and reporting have changed over the course of 10 years, which is the span of this cohort.

### Methodology

• retrospective

• observational

• multicentre study
