# Supplementary material for: Development and multicenter validation of a multiparametric imaging model to predict treatment response in rectal cancer
Source: Eur Radiol. 2023 Jul 14;33(12):8889–98. doi: 10.1007/s00330-023-09920-6 (PMC10667134; doi:10.1007/s00330-023-09920-6)
Supplement: Supplementary file 1 — (DOCX 44 kb) [file 330_2023_9920_MOESM1_ESM.docx]

# Development and multicenter validation of a multiparametric imaging model to predict treatment response in rectal cancer

**Electronic Supplementary Material (ESM)**

# Supplementary Materials A

| **Supplementary Table A1: Overview of main variations in hardware and acquisition protocols in the 9 participating centers** | | | | | | |
| --- | --- | --- | --- | --- | --- | --- |
| **Hardware** | | | | | | |
| **Total number of scanners** | | | | n=25 | | |
| **Total number of scanner models** | | | | n=13 | | |
| **Vendor** | | | | | | |
|  | Philips Healthcare (used in 6 centers) | | | n=10 (incl. 4 different scanner models) | | |
|  | Siemens Healthineers (used in 5 centers) | | | n=12 (incl. 7 different scanner models) | | |
|  | GE Healthcare (used in 2 centers) | | | n=3 (incl. 2 different scanner models) | | |
| **Field strength** | | | | | | |
|  | 1.5 T | | | n=19 | | |
|  | 3.0 T | | | n=6 | | |
| **Acquisition protocols summary** | | | | | | |
| **Total number of T2 protocols*** | | | | n=112 | | |
| **Total number of DWI protocols*** | | | | n=94 | | |
|  | | **T2W-MRI**  **median (range)** | | | **DWI**  **median (range)** | |
| **Parameter** | | **Development cohort** | **Validation**  **cohort** | | **Development cohort** | **Validation**  **cohort** |
| TR (ms) | | 4263  (1573-16738) | 4244  (1573-16738) | | 5200  (2480-11000) | 6000  (949-7900) |
| TE (ms) | | 122  (68-163) | 114  (60-163) | | 76  (54-117) | 88  (37-96) |
| Flip angle (°) | | 130  (90-165) | 150  (90-165) | | 90  (70-180) | 90  (90-90) |
| NSA | | 2  (1-6) | 2  (1-6) | | 5  (1-15) | 4  (2-10) |
| Slice thickness (mm) | | 3  (3-5) | 3  (3-5) | | 5  (2.7-8) | 6  (5-7) |
| In-plane resolution (mm) | | 0.73  (0.31-0.99) | 0.63  (0.29-1.48) | | 1.63  (0.63-3.52) | 1.98  (1.30-2.05) |
| Total number of b-values | | N/A | N/A | | 3  (2-7) | 2  (2-4) |
| Lowest b-value (s/mm^2^) | | N/A | N/A | | 0  (0-100) | 50  (0-50) |
| Highest b-value (s/mm^2^) | | N/A | N/A | | 1000  (600-2000) | 800  (600-1000) |
| NSA: Number of signal averages, T: Tesla, TE: Echo Time, TR: repetition time.  * Acquisitions were grouped as one protocol when acquired within the same center, using the same hardware and field strength with consistent flip angle, NSA, slice thickness, in plane resolution and – in case of DWI – number and type of b-values. | | | | | | |

# Supplementary Materials B

# Part 1 - Data harmonization

**Rationale and method:**

Retrospectively collected multicenter MRI datasets – including our own – are inherently subject to data variations caused in large part by differences in acquisition protocols between centers. Such variations can have a substantial effect on model performance, in particular when using quantitative data derived directly from the images[1]. Aim of this additional analysis was to investigate whether data harmonization using three different previously published methods may improve predictive performance of models incorporating imaging features:

The following methods were tested:

1. Data normalization using a reference organ or tissue[2]: When performing data normalization using a reference organ, the measurement value of the lesion under investigation is divided by the measurement value of a reference organ or tissue within the image. For this study, reference measurements were performed by placing a single-slice region of interest in a single non-suspicious lymph node in the inguinal region for each patient. Normalization was only performed for DWI (due to misalignment of lymph nodes on corresponding T2W images). To accomplish this, ADC maps were normalized by dividing the whole ADC image by the average ADC value of the lymph node, after which normalized tumor ADC features were extracted from the whole-tumor segmentations following the same methodology as described in the main paper.
2. ComBat normalization[3]: The ComBat algorithm is a recently introduced realignment method that aligns all imaging features into a single feature space (i.e. removing center effects) without altering the biological information of interest. The imaging features were first extracted following the methodology in the main paper. Then, these imaging features were normalized for each feature separately using ComBat with complete response and good response status specified as biological covariates and hospital defined as the batch covariate.
3. Mixed effects models[4]: A mixed effect model is a statistical model that takes into account correlations between certain grouped patients (i.e. coming from the same center) by modeling variations between these groups as a random effect. The features as defined in the main paper were used for this analysis. We used the Binomial Bayes Generalized Linear Mixed Model (GLMM) implementation from the Python “statsmodels” (version 0.12.2) package, and defined the model to have a random intercept with a fixed slope. Since a LASSO implementation of the GLMM was not available, we instead used the Maximum Relevance Minimum Redundancy (MRMR) feature selection method to limit the number of features and prevent overfitting (with the optimal number of selected features either 2, 4, 6, 8 or 10).

After applying the data-harmonization methods described above, normalized imaging features were analyzed in combination with the baseline and staging variables using the same methodology described in the main paper for the development cohort, including repeated hold-out cross-validation. The same random seed for the repeated cross-validation was used so that training and evaluation was performed on the exact same patients included in the main multicenter analysis for each method.

**Results and conclusions:**

Results are presented in **Supplementary Table B1**. While for certain variable categories there were slight changes in diagnostic performance (max +-0.05 AUC), overall the effect of the investigated normalization methods was negligible given the wide overlapping confidence intervals. When focusing specifically on the models including quantitative imaging features, no significant increase in diagnostic performance was observed after application of any of the data-harmonization methods.

| **Supplementary Table B1: Average AUC on the development cohort using different normalization methods** | | | | | | | | |
| --- | --- | --- | --- | --- | --- | --- | --- | --- |
| **Normalization method** | **No normalization used (main paper)** | | **Lymph node normalization** | | **ComBat normalization** | | **Mixed effects – Random intercept fixed slope** | |
| **Variable groups and combinations** | **CR (95% CI)** | **GR (95% CI)** | **CR (95% CI)** | **GR (95% CI)** | **CR (95% CI)** | **GR (95% CI)** | **CR (95% CI)** | **GR (95% CI)** |
| Non-imaging | 0.58  (0.49 – 0.66) | 0.53  (0.42 – 0.58) | 0.58  (0.50 – 0.74) | 0.53  (0.43 – 0.59) | 0.58  (0.46 – 0.66) | 0.53  (0.46 – 0.57) | 0.59  (0.50 – 0.61) | 0.56  (0.48 – 0.60) |
| Non-imaging + basic imaging staging  (original reports) | 0.63  (0.55 – 0.70) | 0.52  (0.39 – 0.54) | 0.62  (0.57 – 0.67) | 0.51  (0.39 – 0.53) | 0.63  (0.54 – 0.65) | 0.52  (0.39 – 0.54) | 0.63  (0.56 – 0.67) | 0.57  (0.46 – 0.59) |
| Non-imaging + basic imaging staging  (expert re-evaluation) | 0.66  (0.58 – 0.70) | 0.62  (0.56 – 0.68) | 0.65  (0.61 – 0.72) | 0.62  (0.58 – 0.76) | 0.66  (0.57 – 0.69) | 0.62  (0.55 – 0.65) | 0.66  (0.62 – 0.72) | 0.62  (0.54 – 0.65) |
| Non-imaging + advanced imaging staging  (expert re-evaluation) | **0.69**  **(0.62** – **0.74)** | **0.67**  **(0.62** – **0.73)** | 0.69  (0.63 – 0.74) | 0.67  (0.62 – 0.72) | 0.69  (0.63 – 0.71) | 0.67  (0.62 – 0.72) | 0.69  (0.64 – 0.76) | 0.67  (0.62 – 0.71) |
| Non-imaging + quantitative imaging | 0.59  (0.46 – 0.61) | 0.58  (0.47 – 0.61) | 0.58  (0.47 – 0.66) | 0.58  (0.46 – 0.65) | 0.61  (0.48 – 0.64) | 0.57  (0.49 – 0.64) | 0.63  (0.56 – 0.68) | 0.60  (0.55 – 0.69) |
| Non-imaging + basic imaging staging  (original reports) + quantitative imaging | 0.59  (0.44 – 0.60) | 0.57  (0.44 – 0.59) | 0.59  (0.47 – 0.59) | 0.58  (0.44 – 0.60) | 0.60  (0.46 – 0.61) | 0.56  (0.45 – 0.56) | 0.63  (0.54 – 0.68) | 0.59  (0.51 – 0.65) |
| Non-imaging + basic imaging staging  (expert re-evaluation) + quantitative imaging | 0.63  (0.51 – 0.68) | 0.62  (0.53 – 0.68) | 0.64  (0.57 – 0.68) | 0.62  (0.53 – 0.66) | 0.64  (0.54 – 0.66) | 0.61  (0.52 – 0.66) | 0.65  (0.59 – 0.72) | 0.62  (0.57 – 0.67) |
| Non-imaging + advanced imaging staging  (expert re-evaluation) + quantitative imaging | 0.68  (0.59 – 0.71) | 0.67  (0.61 – 0.72) | 0.69  (0.62 – 0.71) | 0.68  (0.62 – 0.72) | 0.68  (0.61 – 0.71) | 0.67  (0.60 – 0.72) | 0.69  (0.60 – 0.74) | 0.67  (0.63 – 0.72) |
| 95% CI: 95% confidence interval, CR: Complete response, GR: Good response  NB: Confidence intervals are based on the non-studentized pivotal bootstrap method[5] using 200 bootstrap samples | | | | | | | | |

# Part 2 – Single-center analysis

**Rationale and method:**

Image-based prediction studies analyzing single-center study data are known to frequently result in higher predictive performance compared to multicenter study designs. This is likely related to the fact that single center study data are typically more homogeneous after having been acquired on a single scanner or small number of scanners with consistent imaging protocols, and assessed (e.g. staged, measured) by a single or small group of readers using consistent methods.

In an attempt to perform a direct comparison between a single center (homogeneous) versus multicenter (heterogeneous) study design within our own dataset, the full analysis as described in the main paper (with the exception of external validation) was repeated on a homogeneous subset of 67 patients derived from a single center within our cohort (Center 9) scanned with a consistent acquisition protocol (T2W MRI: in-plane resolution: 0.73x0.73mm, slice thickness: 3 mm, TR: 2550-6490, TE:122, flip angle: 120-150, NSA:1-2; DWI MRI: in-plane resolution: 1.75x1.75 mm, slice thickness 5mm, TR:5200-7000, TE 80, flip angle: 90, NSA: 1-6, b-values: 0, 500, 1000)

**Results and conclusions:**

Results are shown in **Supplementary** **Table B2**. Similar to the multicenter results described in the main paper, the best performing model was the model including non-imaging and advanced imaging staging variables to predict a complete response. This model achieved an AUC 0.79 (95% CI 0.42 – 1.00) which was substantially higher than the AUC 0.69 (95% CI 0.57-0.80) achieved in the multicenter development cohort . For the majority of the variable sets, a higher AUC was obtained for the single center analysis compared to the multicenter analysis when predicting a complete response, albeit with wider confidence intervals. For the prediction of a good response, results were more variable and no clear trend towards higher or lower performance was identified for the single versus multicenter analysis.

| **Supplementary Table B2: Single center model performance** | | |
| --- | --- | --- |
| **Feature sets** | **Outcome** | |
|  | **CR (95% CI)** | **GR (95% CI)** |
| Non-imaging | 0.78  (0.63 – 0.89) | 0.52  (0.25 – 0.57) |
| Non-imaging + basic imaging staging  (original reports) | 0.75  (0.59 – 0.90) | 0.60  (0.35 – 0.62) |
| Non-imaging + basic imaging staging  (expert re-evaluation) | 0.74  (0.53 – 0.87) | 0.50  (0.19 – 0.51) |
| Non-imaging + advanced imaging staging  (expert re-evaluation) | **0.79**  **(0.65 – 0.93)** | **0.62**  **(0.44 – 0.74)** |
| Non-imaging + quantitative imaging | 0.53  (0.15 – 0.57) | 0.49  (0.19 – 0.50) |
| Non-imaging + basic imaging staging  (original reports) + quantitative imaging | 0.55  (0.19 – 0.56) | 0.50  (0.22 – 0.51) |
| Non-imaging + basic imaging staging  (expert re-evaluation) + quantitative imaging | 0.58  (0.24 – 0.62) | 0.48  (0.18 – 0.48) |
| Non-imaging + advanced imaging staging  (expert re-evaluation) + quantitative imaging | 0.70  (0.46 – 0.83) | 0.55  (0.32 – 0.60) |
| NB. No external validation performed as analysis was single center. Confidence intervals are based on the non-studentized pivotal bootstrap method[5] using 200 bootstrap samples | | |

**References**

1. Schurink NW, van Kranen SR, Roberti S, et al (2022) Sources of variation in multicenter rectal MRI data and their effect on radiomics feature reproducibility. Eur Radiol 32:1506–1516

2. Koc Z, Erbay G, Karadeli E (2017) Internal comparison standard for abdominal diffusion-weighted imaging. Acta radiol 58:1029–1036

3. Orlhac F, Lecler A, Savatovski J, et al (2021) How can we combat multicenter variability in MR radiomics? Validation of a correction procedure. Eur Radiol 31:2272–2280

4. Kahan BC (2014) Accounting for centre-effects in multicentre trials with a binary outcome – when, why, and how? BMC Med Res Methodol 14:20

5. Carpenter J, Bithell J (2000) Bootstrap confidence intervals: when, which, what? A practical guide for medical statisticians. Stat Med 19:1141–1164

# Supplementary Materials C

**Coefficients of best performing models trained on the development cohort**

The coefficients of the best performing models (i.e. non imaging + advanced imaging staging variables) to predict complete and good response are reported in **Supplementary Table C1.** Since feature centering was applied when training these models (i.e. all features within the development cohort were scaled to have mean=0 and standard deviation=1), the coefficients of the resulting models have been transformed back to their original scale. The centering factors (i.e. the mean and standard deviation) of the development dataset are also reported.

| **Supplementary Table C1: Coefficients of best performing models trained on the development cohort** | | | |
| --- | --- | --- | --- |
|  | **Coefficients**  **(original scale)** | **Mean** | **Standard deviation** |
| **Complete response model:** | | | |
| Intercept | -0.9898 | 1 | 0 |
| Tumor height[Low] | -0.3706 | 0.888 | 0.315 |
| Weeks to surgery | 0.0521 | 10.932 | 3.539 |
| cTsub-stage | -0.2855 | 3.411 | 0.635 |
| cN-stage | -0.2620 | 0.874 | 0.772 |
| Invasion depth (mm) | -0.0832 | 5.704 | 7.271 |
| Tumor length (mm) | -0.0066 | 46.687 | 14.648 |
| **Good response model:** | | | |
| Tumor height[Low] | -0.1620 | 0.888 | -0.315 |
| Weeks to surgery | 0.1458 | 10.932 | 3.539 |
| cTsub-stage | -0.2607 | 3.411 | 0.635 |
| MRF status | -0.0691 | 0.299 | 0.458 |
| Invasion depth (mm) | -0.2621 | 5.704 | 7.271 |
| EMVI status | -0.0767 | 0.383 | 0.487 |
